# When does activism benefit well-being? Evidence from a longitudinal study of Clinton voters in the 2016 U.S. presidential election

**DOI:** 10.1371/journal.pone.0221754

**Published:** 2019-09-05

**Authors:** Patrick C. Dwyer, Yen-Ping Chang, Jason Hannay, Sara B. Algoe

**Affiliations:** 1 Lilly Family School of Philanthropy, Indiana University, Indianapolis, Indiana, United States of America; 2 Department of Psychology and Neuroscience, University of North Carolina at Chapel Hill, Chapel Hill, North Carolina, United States of America; Saint Peter’s University, UNITED STATES

## Abstract

Contrary to the expectations of many, Hillary Clinton lost the 2016 U.S. presidential election. The initial shock to her supporters turned into despair for most, but not everyone was affected equally. We draw from the literature on political activism, identity, and self-other overlap in predicting that not all Clinton voters would be equivalently crushed by her loss. Specifically, we hypothesize that pre-election measures of political activism, and level of self-other identification between participants and Clinton–that is, how much a person was “with her”–will interact to predict the level of distress of Clinton voters two months later. Longitudinal data support our hypothesis. Notably, among Clinton voters, greater activism negatively predicted depressive symptoms, and positively predicted sleep quality, but only when participants were highly identified with Clinton. We discuss the implications of the results for theory and research on social action and well-being.

## Introduction

The 2016 U.S. presidential election was among the most contentious in U.S. history [1]. Political activists of all stripes pulled out all the stops to get their candidate elected, with billions of dollars spent on advertising, and local activists volunteering to make phone calls on behalf of their candidates, even taking on watchdog positions of citizen poll-watchers to ensure a fair election [2, 3]. The two main sides–those in favor of then Republican candidate and now President Donald Trump and those in favor of Democratic candidate Hillary Clinton–framed this as a “last chance”. The stakes were high. For Republicans, Trump represented the last person standing between them and another Obama-like administration. For Democrats, Clinton may have stated the case most clearly when she said to a newspaper reporter, “I’m the last thing standing between you and the apocalypse” [4].

As history shows, Hillary Clinton lost. With the looming inauguration of Trump, many of Clinton’s voters feared for the worst. Surprised and shocked by the election outcome [5], these feelings soon turned to “incredible sadness” and despair among some Clinton voters [6]. As we know from psychological research, some people will respond to such distressing events with an increase in depressive symptoms [7] and sleep disturbances [8]. However, different situational and psychological variables may interact in a way that assists people in their recovery from particularly distressing news. In the current work, we draw from the literatures on political activism, identity, and self-other overlap to predict that, although many felt crushed, some people might recover faster and fare better.

### Activism and well-being

A growing body of research suggests that engaging in political activism can promote well-being [9–17]. This evidence is consistent with the idea, long suggested by psychologists and social theorists, that working toward something bigger than the self is a fundamental human striving, and that the satisfaction of this basic motive promotes well-being [18–20]. It’s also consistent with more recent research showing that actions that are directed toward the welfare of others or one’s community facilitate personal well-being [21].

People appear to be happier and more satisfied with life when they connect up with other individuals, groups, and the larger society, and when they work together to improve society’s functioning. Across multiple studies, including surveys of undergraduate students as well as a national sample of U.S. activists matched with control participants, Klar and Kasser [14] found that activism was positively associated with participants’ well-being. Activists also appear to have a greater sense of self-esteem than non-activists [10]. Moreover, Boehnke and Wong [9] found that failing to take action in the face of a perceived sociopolitical threat leads to a poorer long-term mental health trajectory. Even engaging in activism online (e.g., tweeting about sexism) can benefit well-being [12].

However, as noted by Vestergren, Drury, and Hammar Chiriac [15] in their review of the psychological consequences of activism, these consequences aren’t always positive and can sometimes even be negative. Activists can experience burnout, or a state of emotional exhaustion through their work [22, 23]. For example, in one study [24] this was found to be the case among animal rights activists, who suffered burnout as a result of the failure of their campaigns. Similarly, in the case of supporters of a political candidate, it seems likely that while some may reap psychological benefits from their activism, others may not. This may be especially true after an objective failure of one’s activist efforts, such as when one’s candidate loses an election. Thus, we felt that surveying Hillary Clinton’s supporters in the context of the 2016 U.S. presidential election could provide a valuable opportunity to address the question of when a person’s activism serves to benefit their well-being.

### The role of identity

One slogan of the Clinton campaign was “I’m with her”. Moreover, reports of growing ambivalence toward Clinton as the election drew closer [25, 26] suggest that some Clinton voters were more “with her” than others. In the present research, we consider the extent to which Clinton voters included her in their sense of self, and how this might affect the link between activism and well-being.

Theory on collective action proposes the important role played by an individual’s identity, particularly their social identity, or the part of their self-concept that is based on membership in various social categories [27]. For example, both the Elaborated Social Identity Model [28, 29] and the Dynamic Dual Pathway Model [30] emphasize the dynamic interplay between activism and social identity. An individual may be motivated to engage in activism due to their social identity, and their social identity can also be shaped and strengthened through participation in activism. Moreover, this heightened sense of shared identity can lead to positive psychological outcomes, such as a sense of empowerment [31]. Empirical evidence supports this proposition. In interview studies of activists, Drury, Cocking, Beale, Hanson, and Rapley [32] concluded that activism that “actualizes participants’ social identity against the power of dominant groups” was linked to activists’ accounts of empowerment and positive emotions resulting from their activism (p. 309), and Evripidou and Drury [11] concluded that whether activists experienced positive emotions and defined their activities as a success depended on their social identities, which appeared to have shaped their goals and expectations. Similarly, through interviews with environmental activists, Vestergren et al. [17] concluded that a heightened sense of a shared identity contributed to well-being benefits that were experienced by activists.

Researchers have also begun to use quantitative methods to examine the relations between activism, social identity, and well-being. Foster [13] reasoned that if activism can bolster a person’s social identity, then it might also bolster the positive consequences of social identity in the face of discrimination, namely by enhancing well-being. This study found support for the proposition by making gender discrimination salient, and then experimentally manipulating participants’ online activism against sexism. The work approaches our current goals but we have yet to find a paper that quantitatively tests our core thesis, which recognizes the variety of reasons that activists engage in their activism (i.e., because they believe in a political candidate, or they believe in the cause, or they are *against* the other candidate): does the effect of activism on well-being depend on one’s identity?

The present study builds on previous research in at least three ways. First, this study quantitatively examines the basic interaction between activism and identity on well-being. Second, this study complements Foster’s [13] work, in which measures were collected at a single time point, by using a longitudinal design that tests the interaction between activism and identity in predicting well-being two months later. As Vestergren et al. [15] noted in their review of the literature on psychological consequences of activism, there is a great lack of longitudinal research in the area. Third, this study expands on previous work by considering the implications for well-being of political activism and identification with a specific political candidate. Prior work in this area has focused on group identity. However, as identity theorists have argued, identification with an individual (i.e., “classical identification”, [33]) can be complementary to identification with a group, such that the former is often generalized to the latter [34]. Here, we test whether an interaction between activism and identity will emerge at the level of identification with an individual who is widely recognized as a representative and leader of a social group.

Specifically, based on previous theory and evidence suggesting the interplay between social identity and activism in promoting well-being, in the context of the 2016 presidential election we expect that well-being benefits for Clinton voters will arise from activism only when identification with Clinton is high. As noted above, it seems likely that not all people who cast their votes for Clinton identified with her at that level. Therefore, this account would suggest the following interactional hypothesis:

**Hypothesis:** Among Clinton voters, pre-election activism will promote post-loss well-being only when the voter identifies highly with Hillary Clinton.

### The present investigation

Participants were surveyed the week before the 2016 U.S. presidential election and provided information about their level of political activism in general and self-identification with Hillary Clinton in particular. They were surveyed again in the days immediately following the election as to whom they voted for. These initial assessments were conducted for a separate study, but because the majority of participants voted for Clinton, we took the opportunity to conduct additional follow up assessments that included items assessing their well-being following Clinton’s loss.

Specifically, they completed two additional surveys, the first just before President Trump was inaugurated, and the second 4–10 days after the inauguration. In both follow-up assessments, participants reported the extent to which they experienced depressive symptoms and their overall sleep quality. Because depression has been frequently linked with poor sleep quality in the literature (e.g., see [35] for a review), and because of the unusually combative and taxing nature of the election period [1], we considered both of these constructs as indicators of participants’ well-being in the wake of their candidate’s loss.

## Method

### Procedure

All research activities were approved by the IRB of the University of North Carolina at Chapel Hill. Consent was obtained from all participants. The election’s results presented an opportunity to learn more about how such an event affects mental and physical well-being among those who were involved. To test this, we supplemented an ongoing project that happened to assess general voters once before (T1a; November 3^rd^ to 7^th^, 2016, M = the 5^th^ 11:55 am) and once after (T1b; November 9^th^ to 30^th^, 2016, M = the 11^th^ 8:06 pm) the 2016 U.S. presidential election (November 8^th^, 2016). There was a random-assignment between-participant experimental manipulation at T1a that aimed to induce a self-transcendent positive, a self-focused positive, or a neutral emotional state. However, the manipulation check showed the manipulation was not successful, providing justification for the current longitudinal correlational design. Indeed, as documented in the Appendix, there was no manipulation effect on any of the variables used in the current study. We extended this project for the present research with two more follow-up surveys, one before (T2; January 3^rd^ to 18^th^, 2017, M = the 10^th^ 1:17 am) and the other after (T3; January 30^th^ to February 8^th^, 2017, M = the 1^st^ 4:32 am) the 2017 presidential inauguration (January 20^th^, 2017).

At T1a, participants reported whom they would vote for in the upcoming election, level of identification with their preferred candidate, dispositional activism, and daily emotions experienced in the week before the survey. Here, 86.38% of participants indicated that they would vote for Clinton; for them, the identification question was thus identification with Clinton. After the election at T1b, participants then answered if they voted at all and, if so, for whom; similar to what we found at T1a, a great majority of participants—87.28%—reported that they voted for Clinton. Given the disproportionate support for Clinton and, consequently, a lack of statistical power to represent other voters, we only examined Clinton voters and their levels of post-election well-being—or more precisely, ill-being—once before (T2) and once after Trump was sworn into office (T3).

These last two waves of questionnaires were exactly the same, because we had no a-priori prediction of differences between time points; using exact duplications then practically gave us a better chance to follow up with each participant at least once as participants had not originally consented or committed to follow-ups. For those who did take both follow-ups, the repeated responding also increased their data reliability. Finally, we use a multilevel modeling (MLM) approach to best preserve the response data we received [36, 37]. Specifically, MLM does not require symmetric data structure across participants, as would be required in ANOVA or regression, so we did not have to randomly choose one and then lose the other follow-up when a person had sent in two [38]. We also did not have to average across the two to retain the information from both but then lose the variability between the two (as needed in multiple regression). Unlike these other strategies, MLM uses all information in the data as it is. All four surveys were administered online, and participants were contacted via their email addresses provided at T1a.

### Participants

We received completed part-a surveys from 398 adult participants (including those who did not intend to vote for Clinton; age range = [18.25, 75.83], M = 40.70, SD = 14.68; women = 82.56%, men = 15.80%) at T1a through the informational listserv of the University of North Carolina at Chapel Hill, and 306 of the 398 completed part-b surveys at T1b. Among these 306 participants who finished the entire two-part original study, only 299 (i.e., 97.71%) passed all attention checks at both time points. To ensure response quality, in the questionnaires at T1a, T1b, T2 and T3, we inserted an attention check question: “Which planet are you on now?” with options of the planets in the solar system. We also used a second attention check question at T1a, T2, and T3: “Please scroll to 20.” on a negative-50-to-positive-50 scroll bar. We only invited individuals who passed both attention checks to participate in the follow-up assessments, once at T2 and again at T3, in exchange for a raffle to win an e-gift card for each follow-up survey completed.

Among the 299 individuals invited for the follow-up assessments, 167 from the group responded, followed through on their intention (reported at T1a) to vote for Clinton (reported at T1b), and passed the attention checks installed in the follow-ups at least once at T2 (N = 127) or T3 (N = 131). Three entries from Clinton voters at T2 and 4 entries at T3 failed the attention checks. These follow-ups were excluded from analyses. In addition, when predicting which Clinton voters did vs. did not respond to the follow-ups, three independent logistic regression analyses confirmed that the likelihood of completing a follow-up questionnaire was not significantly predicted by activism (B = 0.01, SE = 0.10, Wald = 0.01, *p* = .917), level of identification with Clinton (B = 0.02, SE = 0.12, Wald = 0.02, *p* = .882), or emotions assessed at T1a (B = −0.20, SE = 0.12, Wald = 2.67, *p* = .102). Based on the suggestions of peer reviewers, we also explored the association between gender and willingness to participate in the follow-ups, and found that gender did predict participants’ willingness to do so. Specifically, women were more willing to take part in the follow-ups (of the original participants, 52.0% completed T2 and 52.9% completed T3) than were men (33.3% and 36.1% respectively; chi-sq = 4.33, *p* = .037 for T2; chi-sq = 3.51, *p* = .061 for T3). We thus added gender as a control variable into the models described below, predicting depressive symptoms and sleep quality, and reran the analyses. However, in both models adding gender did not influence the conclusions described below.

Ultimately, 91 of the 167 qualified Clinton voters participated in both the T2 and the T3 follow-up, so we received a total of 258 completed and attention-checked follow-up responses, i.e., 167 individuals’ first follow-up + 91 second follow-ups (or 127 T2 entries + 131 T3 entries). With their 167 T1 counterparts (one per individual), these 258 follow-up assessments then became the data used in the present investigation.

### Measures

#### Activism

We measured activism via the Activist Identity and Commitment Scale [14]. Past research shows that the scale is not only reliable and valid but also associated with activists’ well-being [14]. In the current study, we administered all eight Likert-type items from the scale, which participants answered on a 0-to-5 scale where a higher score indicated stronger activism, and computed the average of the items. Sample items include “Being an activist is central to who I am.”, and “I make time for activism, even when I’m busy.” Please find the Cronbach’s *α* of this and other measures used in the current study in Table 1.

**Table 1:** Descriptive statistics.

	N	Mean	SD	Skewness	Cronbach’s *α*
Activism	167	2.97	1.33	0.17	.97
ICS	167	2.89	1.11	0.00	n/a
MDESn	167	3.38	1.03	0.05	.84
T2 CESD	127	1.72	0.50	1.03	.92
T2 Sleep disturbance	127	1.24	0.68	0.27	n/a
T3 CESD	131	1.81	0.50	0.76	.91
T3 Sleep disturbance	131	1.24	0.71	0.38	n/a

*Note*: ICS and sleep disturbance have no Cronbach’s *α* because they were single-item measures.

#### Inclusion of Clinton in the self (ICS)

To measure one’s identification with Clinton, we modified the Inclusion of Other in the Self scale [39] by replacing “Other” with the name of whomever a participant reported they would vote for. Because all participants indicated they would vote for Clinton, the assessment was therefore a measure of “Inclusion of Clinton in the Self”. We measured this on a pictorial five-point (1-to-5) Likert-type scale where every point had two separate circles, labeled “Self” and “Clinton” with increasing levels of overlap going up the scale.

#### Center for Epidemiological Studies Depression Scale (CESD)

The CESD is a 20-item self-report Likert-type scale that assesses general and clinical populations’ recent depressive symptomatology [40]. The scale has been widely used in research and has demonstrated high reliability and validity [40]. In the current study, we utilized the CESD as one of two ill-being measures of the potentially distressing effects of the 2016 election and political transition. We administered the assessment with a 4-point scale ranging from experiencing (over the past week) some depressive symptom “rarely or none of the time (less than 1 day; point 1)”, “some or a little of the time (1–2 days; point 2)”, “occasionally or a moderate amount of time (3–4 days; point 3)”, “most or all of the time (5–7 days; point 4)”, and computed the average of the scale items. Sample items include “I felt sad.”, and “I enjoyed life.” (reverse-scored).

#### Sleep disturbance

We assessed subjective sleep quality as another indicator of well-being with the Pittsburgh Sleep Quality Index (PSQI; [41]. As with the CESD, the PSQI is a widely accepted assessment in the literature of mental, physical, and general health, and includes items such as “During the past month, how would you rate your sleep quality overall?” Importantly, the measure is designed to assess sleep “disturbance” so, following its published scoring procedure, the higher the score—ranging from 0 to 3—the more severe the issue and the poorer one’s sleep is. We therefore call the measure sleep *disturbance* as opposed to *quality* throughout simply for clarity. Readers should note that the full PSQI encompasses various specific components of sleep problems ranging from mental to medical issues (e.g., sleep-related substance abuse), which we did not expect to encounter in the current non-clinical sample (e.g., a sudden change toward abusing substances). Thus, we only analyzed the single-item component of subjective overall sleep quality, the component most closely related to global sleep disturbance across a variety of populations [42].

#### Modified Deferential Emotion Scale—Negative (MDESn)

At T1a, we measured well-being using a different measure—the Modified Differential Emotion Scale [43], with a time-course of emotions experienced *over the previous week*. Because our follow-up questionnaires specifically included depressive symptoms and sleep disturbance, to match the measures’ negative valence, we used the negative emotion subscale of MDES as the baseline control variable in our analyses. The subscale included ten Likert-type items that each asked how much one felt a set of three closely related negative emotions in the past week. For example, “How much were you stressed, nervous, or overwhelmed in the past 7 days?” The scale was administered using a 7-point scale with the points 1, 4, and 7 labeled “Not at all”, “Moderately”, and “Extremely”, respectively, and the average of the items was computed.

## Results

### Descriptives

The descriptive statistics in Table 1 indicate that, before the election, participants had moderate levels of activism, ICS, and MDESn. These constructs had reasonable variability and were distributed fairly symmetrically. Importantly, the variables did not significantly correlate with each other (Table 2) and were suitable for the planned investigation of interactions. As for the dependent ill-being measures, the results in Table 1 also indicated that, in the two follow-ups after the election, individuals did report a moderate, if not excessive, level of depressive symptomatology or sleep disturbance. We therefore believed the measures captured the variability needed to test the hypotheses. Finally, correlations in Table 2 showed that the ill-being measures had good internal consistency and predictive validity, in that they were highly correlated with themselves across time points and moderately with each other. With significant and moderate correlations with ill-being indices, MDESn also seemed to be a reasonable baseline approximate to CESD and sleep disturbance.

**Table 2:** Correlations.

	Activism	1	2	3	4	5
1) ICS	.136					
2) MDESn	.123	.069				
3) T2 CESD	−.105	−.064	.348 *			
4) T2 Sleep disturbance	.011	−.058	.237 *	.430 *		
5) T3 CESD	.034	−.026	.497 *	.704 *	.393 *	
6) T3 Sleep disturbance	−.094	−.043	.270 *	.323 *	.680 *	.474 *

*Note*:

* indicates *p* < .05.

### Analytic strategy

Using the maximum likelihood estimator with robust standard errors, we fit the following two-level random-intercept model in which ill-being measures at a time point were predicted by activism, ICS, their interaction, and baseline MDESn:

$$\text{Ill}-{\text{being}}_{\text{ti}}=\gamma_{0}+\gamma_{1}*{\text{DESn}}_{\text{i}}+\gamma_{2}*{\text{Activism}}_{\text{i}}+\gamma_{3}*{\;\text{ICS}}_{\text{i}}+\gamma_{4}*{\text{Activism}\;\text{x}\;\text{ICS}}_{\text{i}}+u_{\text{i}}+r_{\text{ti}},$$

where *t* denotes two time points—T2 and T3—at the within-person level, *i* denotes participants at the between-person level, and all predictors are grand-mean centered. Further, we did not estimate a random slope at the between-person level because the model would saturate with only two time points at the within-person level. Yet we did not choose to analyze the data in mixed ANOVA, because not everyone had completed both T2 and T3 follow-ups and the current modeling would retain more data collected than would ANOVA. Finally, because all explanatory variables were at the between-person level–that is identification and activism—we chose grand-mean centering instead of person-mean centering.

### Results

**Fig 1:**
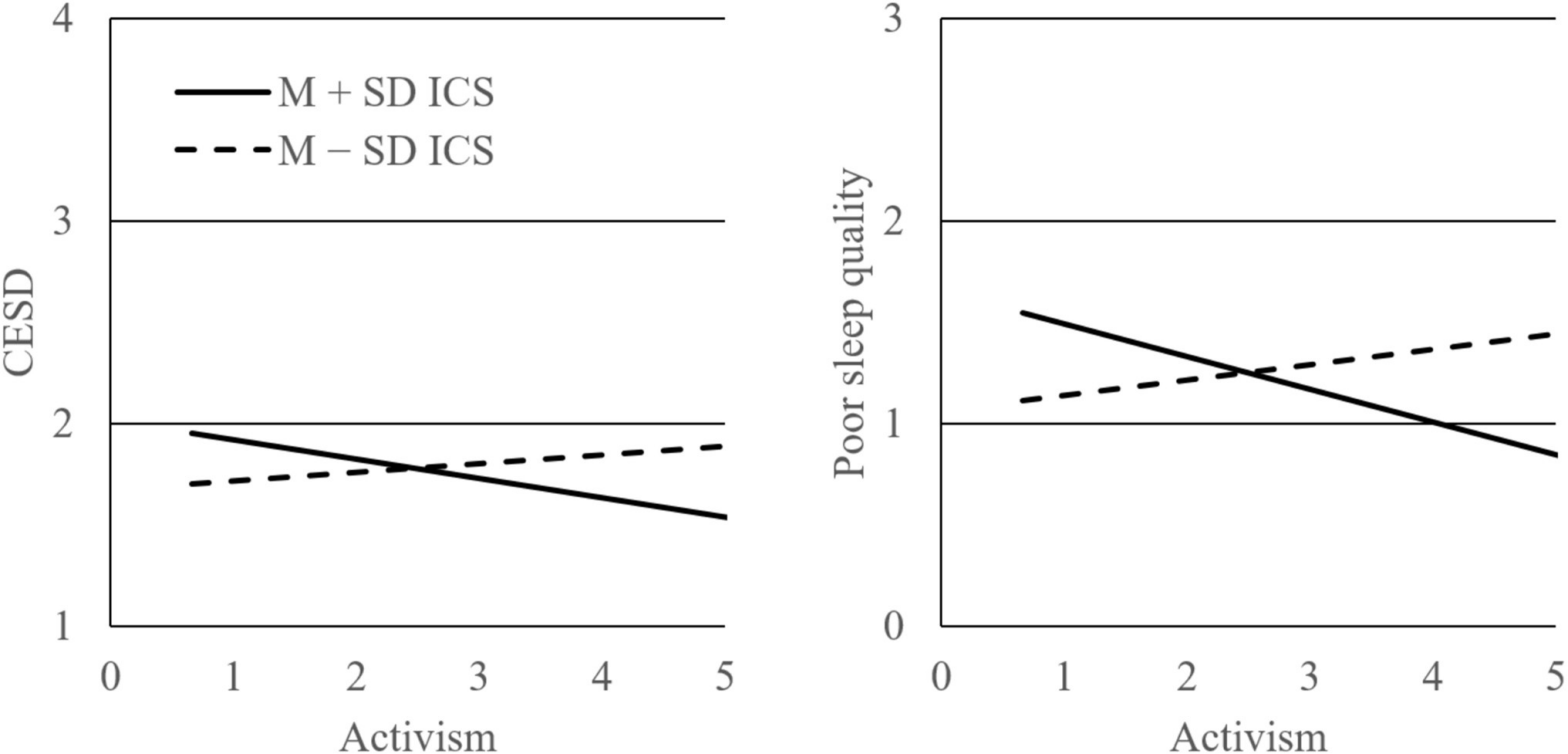
The effects of activism on well-being under ± 1-SD ICS and mean MDESn. Interactions are significant in both graphs; the simple slopes of the solid lines are significant, and those of the dashed ones are not.

Supporting our predictions, the results in Table 3 showed that activism prior to the election was significantly moderated by ICS at the time of the election in predicting both CESD and sleep disturbance two months following the election. Because the main effect of activism was not significant in both models, it was then inferred that ICS reversed the effect of activism on well-being, making activism potentially beneficial for people high in ICS but detrimental for those low in ICS. Looking closely into the interaction, the simple effects (Table 3) visualized in Fig 1 indicated that, regarding CESD, it was high ICS that made activism significantly beneficial and predict lower depressive symptomatology. Low ICS, on the other hand, actually made activism non- to negatively related with CESD. This negative effect nonetheless was not significant. As for sleep disturbance, the same pattern emerged in that, under high ICS, activism significantly predicted less sleep disturbance, but activism did not significantly predict sleep disturbance under low ICS.

**Table 3:** Results of main analysis.

Predictor	*γ*	SE	*df*	*t*	*p*	CI 95% bounds	
						Lower	Upper
CESD							
Intercept *	1.77	0.03	166.07	54.62	.000	1.70	1.83
MDESn *	0.21	0.03	168.56	6.62	.000	0.15	0.27
Activism	−0.03	0.02	168.35	−1.12	.266	−0.08	0.02
| M + SD ICS *	−0.09	0.03	164.10	−2.52	.013	−0.16	−0.02
| M − SD ICS	0.03	0.03	167.26	0.98	.330	−0.03	0.10
ICS	−0.03	0.03	160.33	−0.90	.367	−0.08	0.03
| M + SD Activism *	−0.10	0.04	162.41	−2.42	.017	−0.18	−0.02
| M − SD Activism	0.05	0.04	160.21	1.14	.255	−0.03	0.12
Activism x ICS *	−0.05	0.02	162.44	−2.59	.010	−0.10	−0.01
Sleep disturbance							
Intercept *	1.23	0.05	163.49	25.45	.000	1.14	1.33
MDESn *	0.16	0.05	165.79	3.31	.001	0.06	0.25
Activism	−0.04	0.04	165.59	−0.99	.325	−0.11	0.04
| M + SD ICS *	−0.14	0.05	161.68	−2.59	.010	−0.24	−0.03
| M − SD ICS	0.06	0.05	164.59	1.25	.212	−0.04	0.16
ICS	−0.04	0.04	158.20	−0.89	.373	−0.12	0.05
| M + SD Activism *	−0.16	0.06	160.13	−2.58	.011	−0.28	−0.04
| M − SD Activism	0.08	0.06	158.08	1.33	.187	−0.04	0.20
Activism x ICS *	−0.09	0.03	160.15	−2.85	.005	−0.15	−0.03

*Note*:

* indicates *p* < .05;

^|^ indicates simple effects under a given condition.

## Discussion

The buildup to the 2016 U.S. presidential election suggested it was a once-in-a-lifetime event, both in the news media and by the candidates and their supporters [44]. In the end, Trump’s victory crushed the hopes of many of Clinton’s supporters. The unusually combative and high-stakes nature of this election, along with our pre-election sample consisting largely of Clinton voters, offered the rare opportunity to examine social psychological factors that may predict outcomes relevant to individuals’ well-being, specifically depressive symptoms and sleep quality, after a large-scale societal event. The results of the current longitudinal study, which build on prior literature, revealed an important moderator of the influence of activism on both of these outcomes. Specifically, for Clinton voters, activism negatively predicted depressive symptoms, and positively predicted sleep quality, but only when participants were highly identified with her. When participants were low in their identification with her, activism was not significantly related to either well-being outcome.

We feel there are a number of potential explanations for this pattern of results. First, theory and research suggest participation in collective action can be driven by different motives, such as building solidarity among group members or expressing personal values, which can operate separately from beliefs about how effective action will be at bringing about change [45, 46]. If those highly identified with Clinton were acting for solidarity-building reasons, they may have garnered benefits through a sense of community with other Clinton supporters. This explanation is suggested by an interview study of environmental activists, which concluded that changes in their well-being appeared to emerge out of a new sense of shared identity with other activists, which they felt promoted a sense of community [17]. Or, those highly identified with Clinton may have been acting for value-expressive reasons, which can also have positive consequences for well-being. For example, research suggests that when engaging in activism is consistent with an individual’s values and goals, the more personally significant they feel as a result [47].

In addition, activists who were highly identified with Clinton may have simply reinterpreted the election loss in a more positive way. Theory and research on identity and activism suggest that strong group identifiers utilize reappraisal as a coping resource, such that losses may be reinterpreted as gains [30, 31, 48], in the service of maintaining a positive view of the self. Related to this possibility, recent research has shown that Clinton voters who used reappraisal in response to viewing Trump-oriented news footage reacted to the video with less negative emotion [49]. A similar process may have taken place among activists who were highly identified with Clinton in the present study.

Furthermore, activists who were highly identified with Clinton may have been more passionate about their work. Vallerand et al. [50] define passion “as a strong inclination toward an activity that people like, that they find important, and in which they invest time and energy” (p. 757). An activity must also be part of one’s identity in order to be a passion [50, 51]. Moreover, Vallerand and colleagues have proposed two types of passion, harmonious and obsessive. Whereas harmonious passion has been found to promote psychological well-being, obsessive passion has been found to promote anxiety and depression [51, 52]. And, among activists, harmonious passion has been more closely linked to positive emotions [53] and to less defensiveness in identity-threatening situations [54]. So another possible reason why participants who scored high on activism, and who also strongly identified with Clinton, reported greater well-being may be because they had high levels of harmonious passion for the work they were involved in.

### Implications

Interestingly, and somewhat surprisingly, we found no main effect of political activism on either well-being outcome. This finding is at odds with previous work finding that activism on its own predicted well-being (e.g., [14]). This could be due to the negative nature of the event we considered (i.e., the election defeat). Had Clinton won the election, then perhaps activism alone (i.e., independent of identification with Clinton) would have benefited well-being because activists with a broader range of motives would have seen their motives satisfied. For instance, activists who supported Clinton just because they wanted to see Trump *not* get elected would be happy. Relatedly, the possibility that many participants were not Clinton supporters to begin with, but simply ended up voting for her because she was the Democratic nominee, could also explain the lack of an independent effect of activism. Instead, however, we feel that we found something more interesting in the psychological space where activism intersected with identity. Greater identification with Clinton appeared to unlock the benefits of political activism on well-being, both with regard to depressive symptoms and sleep quality. Only those activists who were firmly “with her” going into the election were protected from depression and sleep disturbance following her loss.

These findings have implications for theory and research on the benefits of social action for the individuals who engage in it. Specifically, they speak to the literature linking political activism to a person’s well-being. Previous work in this area suggests that being dedicated to fighting for something larger than the self can promote personal well-being [14, 55]. This is consistent with a growing body of work showing that doing good for others, more generally, promotes health and happiness (e.g., [56–58]). Though fighting for a cause larger than one’s self may have personally beneficial effects, failures or momentary setbacks can in turn have negative effects on well-being [15]. This research helps shed light on contextual factors that predict when the potentially deleterious effects of failure can be mitigated. The current findings provide some support for self-other inclusion as a situational factor that can help mitigate the deleterious effects disappointment, setbacks, or failure have on personal well-being when one is fighting for a political cause larger than the self.

Identifying with a political candidate has implications for activists’ personal outcomes. Moreover, by showing that activists who highly identify with their chosen candidate are better off in the end, this work contributes to understanding the potential power of identifying with the person you are working to help, more generally. Thus, looking beyond activists to others who work for a cause larger than themselves, this work also suggests that volunteers and others who engage in collective action may experience greater self-benefits when they strongly identify with the recipients of their services. This possibility is consistent with research showing that self-benefits associated with volunteering are particularly likely to arise when a person engages in volunteerism for other-oriented, rather than self-oriented, reasons [59, 60]. Connecting with those on the receiving end of one’s actions appears necessary for self-benefits to arise. Moreover, considering that the recipients of volunteer services are often members of socially marginalized groups (e.g., immigrants, the homeless, people with mental disabilities), which may deter potential volunteers who may fear being stigmatized through their service (e.g., see [61]), recruiting and retaining potential volunteers who are highly identified with service recipients may be critically important for organizations that rely on the efforts of volunteers.

The current work also provides insights that could be useful to individual activists or organizations that rely on activists in dealing with negative experiences that arise. Specifically, it suggests a potential path toward forecasting, based on reports of specific psychological constructs, which activists are more likely to bounce back after a setback, and which are less likely to do so. Additionally, it provides recommendations as to which people well-being interventions might most effectively be directed. Efforts to intervene by boosting identification with a political candidate, for example, will likely produce greater well-being in some supporters than others. Experimental research aimed at manipulating identification with a political candidate would be useful in determining the utility of this kind of intervention.

### Limitations and future directions

There are other new directions that the current research might contribute to going forward, as well as limitations to this research that we must acknowledge. First, we would like to see these findings replicated, and especially those concerning sleep quality, which was assessed with a single item. Second, due to the correlational nature of the current study, we feel that it would be helpful to test these dynamics using an experiment that manipulates identification with a political candidate, or with an individual who is on the receiving end of help. Third, we wonder about other ways that identification might be involved in activism and political outcomes. For example, are there aspects of the political process that serve to undermine identifying with political candidates, which might unintentionally set up activists to experience distress following setbacks they may encounter down the line? These are all areas where we feel future research would help expand on these dynamics.

Lastly, like all longitudinal studies, the current study suffered from participant attrition and thus potential self-selection bias. However, we do not think it affected the current results substantially because we were not looking at the mean level change in any variable. Instead, this research focused on how two variables—activism and identification—together predict post-election well-being. Here, we feel selection bias is less likely because it would have to involve a third variable that makes the interactive pattern that we observed appear in the current group of participants but not the group that dropped out.

## Conclusion

The 2016 U.S. presidential election was among most contentious in U.S. history. Despite what most polls predicted, Hillary Clinton lost, and this shocking news soon turned to despair among some of her supporters. In the same way that it was a once-in-a-lifetime election for voters, it presented a unique opportunity to use a significant social and political event to help further our understanding of when activism serves to benefit well-being. Drawing from the literature on political activism, identity, and self-other overlap, we hypothesized that being “with her”–identifying with Hillary Clinton–and level of political activism would interact to predict the level of distress of Clinton voters two months later. The results of this longitudinal study supported this hypothesis in that, among Clinton voters, activism negatively predicted depressive symptoms, and positively predicted sleep quality, but only when participants were highly identified with Hillary Clinton. That is, activism predicted which Clinton voters fared better after her loss, but only among those individuals who were firmly “with her”.

## Appendix

### T1a manipulation effect

We had no prediction that the manipulation at T1a would influence the variables we focus on in the current study. Nonetheless, to formally examine whether the manipulation at T1a influenced the variables included in the current study, we conducted an exploratory ANOVA in which the three groups of the manipulation were compared on each of the variables under consideration in the current study. The results in Table 4 showed that none of the variables significantly differed between groups. We thus ignored the manipulation and conducted the current study as a longitudinal correlational study.

**Table 4:** Effects of preexisting experimental conditions.

Condition	1		2		3		F	*p*
Variable	M	SD	M	SD	M	SD		
T1a (N = 167)								
MDESn	3.38	1.14	3.31	1.07	3.43	0.89	0.19	.826
Activism	3.09	1.34	2.85	1.45	2.94	1.20	0.45	.636
ICS	2.78	1.19	3.12	1.03	2.81	1.07	1.52	.222
T2 (N = 127)								
CESD	1.76	0.54	1.71	0.44	1.68	0.51	0.25	.781
Sleep disturbance	1.35	0.66	1.09	0.56	1.23	0.78	1.50	.227
T3 (N = 131)								
CESD	1.84	0.54	1.87	0.50	1.72	0.47	0.97	.380
Sleep disturbance	1.36	0.67	1.08	0.73	1.27	0.73	1.82	.166

## Supporting information

S1 File Study dataset. (SAV)
